# Lunotriquetral Synostosis as a Cause of Ulnar Sided Wrist Pain: A Case Report

**DOI:** 10.5704/MOJ.2403.019

**Published:** 2024-03

**Authors:** MPT Alves

**Affiliations:** Department of Orthopaedic Surgery, Hospital Doutor José Maria Grande, Portalegre, Portugal

**Keywords:** lunotriquetral synostosis, carpal coalition, ulnar sided pain, Minnaar classification

## Abstract

The diagnostic workout of ulnar sided wrist pain may be challenging, since there can be many different causes for it, varying from ulnar nerve problems to fractures. Congenital lunotriquetral synostosis may present as a source of pain in some cases, but it is a rare diagnosis. The author presents a case of post-traumatic ulnar sided wrist pain in a patient with Minnaar’s type 1 congenital lunotriquetral synostosis.

## Introduction

The etiologic determination of ulnar sided wrist pain isusually a challenging work-out. This condition may be acute or chronic and has many differential diagnostics, such as ligament injuries, carpal bone fractures, osteoarthritis and compressive neuropathies. The orthopaedic surgeon must have a thorough knowledge of the anatomy of the wrist and be able to perform a complete and methodical physicalexamination, coupling this with provocative tests to further define the differential diagnosis^1^. The most common imaging evaluation of ulnar-sided wrist pain is the plain radiograph (usually in two planes, anteroposterior and lateral) andmagnetic resonance imaging (MRI). Nowadays, wrist arthroscopy is the most important tool in the diagnosis and management of ulnar sided intra-articular wrist pathology^2^.

Carpal coalition is a rare developmental anomaly, and its incidence is 0.1% and 1.6% in white and African American populations, respectively. There are four types, according to Minnaar: type 1 - proximal pseudoarthrosis (2% of thecases); type 2 - proximal bony bridge (22%); type 3 -complete fusion (75%); type 4 - complete fusion associated to other carpal deformities (1%). According to some authors, the slight movement between the lunate and the triquetrum in Minnaar ’s type 1 is the most capable of provoking ulnar sided wrist pain^3^, especially after trauma. The weaker fibrocartilaginous coalition appears to be more susceptible to stress or trauma.

The author presents a rare case of a Minnaar‘s type 1congenital synostosis between the lunate and the triquetrumbones as a source of ulnar sided wrist pain.

## Case Report

A 25-year-old male presented to the acute trauma unit in June 2022 with a history of a fall over his outstretched wrist, resulting in ulnar sided wrist pain. The patient was previously healthy and never had wrist pain before thetrauma.

The clinical examination was performed. Slight swellingover the dorsal ulnar side of the wrist was observed. A complete physical examination work-out of the wrist wasperformed. No clinical signs of triangular fibrocartilagecomplex (TFCC) lesion, no deformities; tests for ulnarimpaction were negative. Pain was elicited over the Luno-triquetral joint, with painful squeeze test.

**Fig 1:**
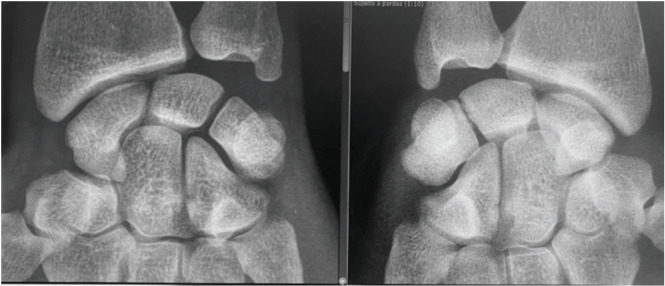
Plain comparative radiograph showing congenital Luno-triquetral synostosis on the right.

The radiograph (Fig. 1) showed a synostosis between the lunate and the triquetrum bones, compatible with Minnaar’s type 1 Luno-triquetral synostosis. Luno-triquetral type 1 synostosis resembles a pseudarthrosis with irregular sclerotic margins. There is a narrowed joint space between the lunate bone and triquetral bone, possibly with subcortical cysts.

Since the patient had no other clinical symptoms of other possible traumatic aetiologies for his pain, no other imaging exams were performed. Treatment consisted of a wrist splint and a non-steroidal anti-inflammatory drug. The patient was followed-up two weeks after trauma. Complete resolution of pain was observed in this assessment and the patient was discharged.

After one year and a half from the initial trauma, in December 2023, the patient was called again for a follow-up visit. He remained asymptomatic and with complete range of motion of the affected wrist.

## Discussion

The identification of the cause of pain on the ulnar side of the wrist can be challenging and the outcome and recovery following surgery can be unpredictable. The ulnar wrist has a complex morphology with a number of delicate structures that can be injured in a traumatic event, thus a wide variety of pathologic conditions can occur. To successfully diagnose and treat these pathologic conditions, the attending orthopaedic surgeon must have a strong understanding of the anatomy and the advanced imaging and diagnostic techniques available^1,2^.

The anteroposterior and lateral radiograph images are usually sufficient to verify a diagnosis of Luno-triquetral synostosis. But in painful cases, other methods, such as CT scan, magnetic resonance imaging and wrist arthroscopy may be indicated^1,2,4^.

The patient had a Minnaar’s type 1 Luno-triquetral synostosis, which led him to have wrist sided wrist pain after trauma; previously, he reported no symptoms. After complete physical examination and exclusion of other conditions, the author opted for conservative treatment. The patient was followed-up in outpatient clinic and at two weeks he was asymptomatic, being discharged of further appointments. If conservative therapy failed, the author considered the surgical management with Luno-triquetral arthrodesis, which has been proven as an effective treatment^3^. As the patient was observed again in a late follow-up visit and remained asymptomatic, no other treatment was considered.

Ou Yang *et al*^1^ reported on 17 different diagnoses on 110 consecutive patients with ulnar sided wrist pain. The most common conditions were TFCC rupture, ulnocarpal impaction, distal radioulnar joint osteoarthritis and extensor carpi ulnaris tendonitis. These conditions had similar clinical symptoms and signs and required either MRI or CT scan for the diagnostic confirmation. The ulnocarpal stress test and the ulnar foveal sign were not sufficiently specific in their series. In their work, there was no mention of Luno-triquetral synostosis as a possible diagnosis.

A few papers in literature^2-4^ mention this condition as a possible cause of ulnar sided wrist pain, turning this into one of the few reports of Minnaar’s type 1 Luno-triquetral synostosis as an etiologic condition for ulnar sided wrist pain. Davis^4^ states that the presence of lunotriquetral coalition and ulnar impaction syndrome in the same wrist is rare, deriving from the fact that lunotriquetral coalition is only present in less than 2% of the population. Sometimes, ulnar impaction syndrome may occur in association with the isolated form of lunotriquetral coalition or as part of a syndrome, most commonly in Minnaar’s type 4, where the wrist presents with other congenital abnormalities. Sometimes, even in cases that present with more than one abnormality in the carpal bones, the patient may be asymptomatic.

Not rarely the finding of a lunotriquetral congenital coalition is incidental. Ozyurek *et al*^5^ found this in a wrist trauma case in which a plain anteroposterior radiograph of left wrist showed a left-sided osseous fusion of the lunate and triquetrum with a distal notch according to Minnaar’s classification type II. Also previously asymptomatic as in the presented case and with the same treatment as ours. Their patient became asymptomatic and returned to normal activities within five days.

In some cases, ulnar sided wrist pain related to Minnaar’s type 1 synostosis can remain symptomatic and resistant to non-operative treatments. Although it did not happen in the presented case, it is worthy to note that he was informed about the surgical possibilities. Operative management of lunotriquetral coalition in such cases has historically been an arthrodesis. As noted by Chiri and Bain^2^ this procedure may have a high complication rate and reduce wrist range of motion. These authors recommend the arthroscopic resection of the synostosis. The advantage of this technique is the fact that they attempt to restore normal anatomy and wrist and carpal kinematics and do not destabilise the joint; however, the authors acknowledge there is a possible risk when performing this procedure and care must be taken not to over-resect.

The knowledge of this congenital anomaly and its possible types of treatment may avoid unnecessary and expensive exams and reduce costs of healthcare. The orthopaedic surgeon should consider this condition in the differential diagnosis when a patient presents with persistent or traumatic ulnar sided wrist pain.
